# Contrast-Associated Acute Kidney Injury and Mortality Risk After Coronary Angiography for Acute Coronary Syndromes: A Retrospective Cohort Study

**DOI:** 10.3390/jcm15093534

**Published:** 2026-05-06

**Authors:** Eva Maria Olivas-Flores, Alberto Daniel Rocha-Muñoz, Angelita del Socorro Valencia-López, Roberto Rojas-Castillo, Jorge Guillermo Delgado-Gutiérrez, Karina Patricia Pizarro-Gonzales, Mario Alberto Mireles-Ramírez, J. Ahuixotl Gutiérrez-Aceves, Nicte Selene Fajardo-Robledo, Adrian Esau De La Cruz-Estrella, Claudia Lorena Mariscal-Chavez, Maria Luisa Vazquez-Villegas, Fabiola Gonzalez-Ponce, Ernesto German Cardona-Muñoz, David Cardona-Müller, Jorge Ivan Gamez-Nava, Laura Gonzalez-Lopez

**Affiliations:** 1 Departamento de Anestesiología, Hospital de Especialidades, Centro Médico Nacional de Occidente, Instituto Mexicano del Seguro Social, Guadalajara 44340, Jalisco, Mexico; eva.olivas@academicos.udg.mx (E.M.O.-F.); angelitavala@hotmail.com (A.d.S.V.-L.); 2 Departamento de Ciencias de la Salud, Centro Universitario de los Valles, Universidad de Guadalajara, Guadalajara 46600, Ameca Jalisco, Mexico; 3 Departamento de Salud-Enfermedad Como Proceso Individual, Centro Universitario de Tonalá, Universidad de Guadalajara, Guadalajara 44100, Jalisco, Mexico; alberto.rocha@academicos.udg.mx; 4 Departamento de Hemodinámica, Hospital de Especialidades, Centro Médico Nacional de Occidente, Instituto Mexicano del Seguro Social, Guadalajara 44340, Jalisco, Mexico; drroca77@gmail.com (R.R.-C.); gdelgado_ii@me.com (J.G.D.-G.); kpizarrog@hotmail.com (K.P.P.-G.); esaugdl@yahoo.com (A.E.D.L.C.-E.); claudiamariscal2014@gmail.com (C.L.M.-C.); 5 División de Educación e Investigación en Salud, Hospital de Especialidades, Centro Médico Nacional de Occidente, Instituto Mexicano del Seguro Social, Guadalajara 44340, Jalisco, Mexico; mario.mirelesr@imss.gob.mx (M.A.M.-R.); ahuixotl@gmail.com (J.A.G.-A.); 6 Laboratorio de Investigación y Desarrollo Farmacéutico, Centro Universitario de Ciencias Exactas e Ingenierías, Universidad de Guadalajara, Guadalajara 44430, Jalisco, Mexico; nicte.fajardo@academicos.udg.mx; 7 Instituto Regional de Investigación en Salud, Departamento de Salud Pública, Centro Universitario de Ciencias de la Salud, Universidad de Guadalajara 44340, Jalisco, Mexico; luisa.vazquez@academicos.udg.mx; 8 Instituto de Terapéutica Experimental y Clínica, Programa de Doctorado en Farmacología, Departamento de Fisiología, Centro Universitario de Ciencias de la Salud, Universidad de Guadalajara, Guadalajara 44340, Jalisco, Mexico; fabiola.gonzalez@academicos.udg.mx (F.G.-P.); cameg1@gmail.com (E.G.C.-M.); david.cardona@academicos.udg.mx (D.C.-M.)

**Keywords:** acute coronary syndrome, coronary angiography, contrast-associated acute kidney injury, stress hyperglycemia, short-term mortality

## Abstract

**Background/Objectives:** Contrast-associated acute kidney injury (CA-AKI) is a frequent complication after coronary angiography (CAG) that may adversely affect outcomes in patients with acute coronary syndrome (ACS). We aimed to estimate the incidence of CA-AKI and evaluate its association with 30-day all-cause mortality in adults with ACS undergoing CAG. **Methods:** We conducted a retrospective cohort study; CA-AKI was defined as an increase in serum creatinine ≥0.5 mg/dL or ≥25% from baseline within 72 h after contrast exposure, according to KDIGO criteria. The primary outcome was 30-day all-cause mortality. Survival analyses were performed using Kaplan–Meier curves and Cox proportional hazards models. **Results:** Including 374 consecutive adults with ACS who underwent diagnostic or therapeutic CAG at a tertiary referral center. The mean age was 68.8 ± 11.2 years, and 72.6% were male. CA-AKI occurred in 17.4% of patients, and 11.7% died within 30 days. In multivariable analysis, age (HR 1.04; 95% CI 1.00–1.07), CA-AKI (HR 2.81; 95% CI 1.48–5.33), stress hyperglycemia ≥180 mg/dL (HR 2.88; 95% CI 1.54–5.38), and delirium (HR 7.20; 95% CI 2.40–20.92) were independent predictors of mortality. **Conclusions:** age, CA-AKI, stress hyperglycemia, and delirium independently predict short-term mortality after CAG in ACS, supporting integrated risk-stratified peri-procedural management. These observations suggest that mortality in these patients may be related to an inflammatory process secondary to ischemia/reperfusion, which is probably induced by dysregulation of the central autonomic network and activation of the hypothalamic–pituitary–adrenal axis which is currently underdiagnosed.

## 1. Introduction

ACS involves a reduction in blood flow due to obstruction of the coronary arteries from rupture of an atherosclerotic plaque, leading to myocardial ischemia or necrosis [1]. The syndrome includes ST-segment elevation myocardial infarction (STEMI), non-ST-segment elevation myocardial infarction (NSTEMI), unstable angina, and troponin elevation [1,2]. CAG is the gold standard test for diagnosing ACS, as it is used to evaluate the percentage of obstruction of the coronary artery and angiographic blood flow (TIMI) [2]. During the CAG, a percutaneous coronary intervention (PCI) with coronary stent placement can be performed [3,4], but several minor or major complications can occur [5]. The major complications include coronary artery dissection and/or perforation, arrhythmia, reinfarction, cardiogenic shock, stroke, and acute kidney injury [5]. CA-AKI is defined as an increase in serum creatinine ≥0.5 mg/dL or ≥25% from baseline within 72 h after contrast exposure [6] and is considered a serious complication of CAG, leading to temporary or permanent kidney damage [7,8]. Some authors have investigated the incidence of CA-AKI after CAG. In a meta-analysis, Lun et al. found that the pooled incidence of CA-AKI after CAG can be as high as 12.8% after the CAG procedures (95% CI: 11.7–13.9%), and the incidence of short-term mortality in patients who developed CA-AKI reached 20.2% (95% CI 10.7–29.7%) [9]; this incidence of CA-AKI-associated mortality did not seem to decline over time [9]. A significant number of studies report a CA-AKI frequency of around 1 in 10 procedures, depending on patient characteristics, comorbidities, and procedural factors [10,11,12,13,14,15,16].

However, the incidence of mortality related to CA-AKI after CAG in specific subgroups, such as patients with ACS, and in patients with different risk factors, needs to be studied in detail while controlling for other variables in multivariate models. Therefore, in a cohort study of adults with ACS undergoing CAG in a specialized coronary care center, we evaluated the frequency of CA-AKI after CAG and the risk of 30-day all-cause mortality.

## 2. Materials and Methods

### 2.1. Study Design and Setting

We conducted a single-center retrospective cohort study of consecutive adults with ACS who underwent diagnostic or therapeutic CAG between January and October 2023 at the Department of Hemodynamics, Hospital de Especialidades, Centro Médico Nacional de Occidente (HE-CMNO), Guadalajara, Jalisco, Mexico. HE-CMNO is a tertiary referral hospital within the Instituto Mexicano del Seguro Social (IMSS), the largest public healthcare system in Mexico. As the principal tertiary referral center for IMSS in Western Mexico, HE-CMNO provides high-complexity cardiovascular care and receives referrals for specialized services from IMSS affiliates in Jalisco, Nayarit, Colima, and Michoacán [17]. The coronary angiography was performed on the Phillips Allura Xper FD20, originally from the Netherlands.

### 2.2. Population and Eligibility Criteria

We included consecutive adult patients (≥18 years) admitted with a confirmed diagnosis of ACS—STEMI, NSTEMI, or unstable angina according to the 2025 ACC/AHA guideline criteria [2]. Eligible patients underwent diagnostic or therapeutic CAG during hospitalization between January and October 2023.

Patients were excluded if they had end-stage kidney disease requiring chronic dialysis before admission; a lack of relevant information, such as changes in serum creatinine measurements required for CA-AKI diagnosis (baseline before angiography and at least one measurement within 72 h after contrast exposure); or insufficient clinical information or insufficient description of angiographic procedures and results for outcome ascertainment.

### 2.3. Study Development and Data Collection

Clinical data were retrospectively extracted from electronic and paper clinical charts for all eligible participants. Data was collected at the time of hospitalization, during CAG, and during the in-hospital stay at every medical visit during the 30 days after the procedure. A standardized electronic case report form (eCRF) was developed to ensure consistent variable definitions and uniform data captured among reviewers. Two trained investigators (E.M.O.F. and N.F.R.) independently reviewed all the charts, laboratory reports, and catheterization records.

Collected variables included (1) demographics and lifestyle factors (age, sex, body mass index, smoking, and alcohol consumption); (2) comorbidities (hypertension, diabetes mellitus, obesity, chronic kidney disease, dyslipidemia, ischemic heart disease, chronic obstructive pulmonary disease, and asthma); (3) clinical characteristics (ACS subtype); (4) angiographic characteristics (contrast volume, revascularization strategy, TIMI flow grade, and duration of the procedure); and (5) laboratory parameters obtained before and after the procedure (complete blood count (hemoglobin, leukocyte count, and platelets count), glucose, urea, serum creatinine, and glomerular filtration rate).

All data were anonymized and stored in a secure institutional database. Data quality was assessed by randomly re-verifying 10% of entries against the source record with an independent reviewer, focusing on key exposures, outcomes, and time-stamped laboratory values.

### 2.4. Structured Assessment and Operational Definitions

All the variables were systematically collected and verified using a standardized eCRF developed for this study. The dataset included the following: (1) ACS, classified as STEMI, NSTEMI, or unstable angina, was evaluated according to the 2025 ACC/AHA guideline criteria [2]. (2) CA-AKI, defined as either an increase in serum creatinine ≥0.5 mg/dL or a ≥25% increase from baseline within 72 h after contrast exposure, and it was evaluated following the Kidney Disease: Improving Global Outcomes (KDIGO) 2012 criteria [6]. Baseline creatinine corresponded to the most recent measurement within 7 days before angiography. (3) Functional status at admission was evaluated using the New York Heart Association (NYHA) classification, while preprocedural risk was assessed using the GRACE score when available. (4) Laboratory parameters (hemoglobin, leukocyte count, platelets, glucose, urea, and creatinine) were analyzed from standardized laboratory reports using institutional reference ranges. (5) Mortality for STEMI or NSTEMI patients due to cardiovascular complications, neurologic injury, and procedure-related mortality was recorded within 30 days after CAG, verified by cross-check with other hospital records and pathology registry data.

### 2.5. Endpoints

The primary endpoint was 30-day all-cause mortality, defined as death from any cause occurring within 30 days after CAG (day 0), ascertained from hospital records and available follow-up documentation. Trained researchers reviewed hospital records (both electronic and paper charts) to identify deaths occurring during hospitalization. In this 30-day survival study, patients were considered censored if they did not experience the event of interest (death) within the 30-day window, or if their outcome status was unknown at the end of that period. To exclude 30-day mortality in those patients who were discharged, the electronic medical record system of the National Social Security System (Mexican Social Security Institute) was reviewed. We did not collect information by phone follow-up or direct communications with patients or their families

Secondary endpoints included the following: (1) CA-AKI, defined as an absolute increase in serum creatinine ≥0.5 mg/dL or a relative increase ≥25% from baseline within 72 h after exposure to iodinated contrast media, according to the Kidney Disease: Improving Global Outcomes (KDIGO) 2012 criteria [6]. Baseline creatinine was the most recent value obtained within 7 days before angiography. Post-procedure creatinine values were obtained at approximately 24, 48, and 72 h after contrast exposure. (2) In-hospital outcomes, including length of stay, new initiation of dialysis during the hospitalization, and intensive care unit admission.

### 2.6. Statistical Analyses

Continuous variables are expressed as mean ± standard deviation (SD), whereas categorical variables are summarized as absolute frequencies and percentages. Group comparisons were performed using Student’s *t* test or the Mann–Whitney U test for continuous variables and the chi-square or Fisher’s exact test for categorical variables, as appropriate. Survival probability at 30 days was estimated using Kaplan–Meier curves, and differences between groups were evaluated with the log-rank test. Event (Uncensored): Patient dies on days previous to 30-day follow-up. Two censored were considered: (a) Censored: Patients who were alive at the end of the study on day 30. (b) Censored: Patients who were discharged (lost to follow-up) before 30 days and did not die (absence of death in the registers), but no additional information was obtained. Univariate Cox proportional hazards regression was performed to identify potential predictors of 30-day all-cause mortality. Variables with a *p*-value < 0.20 in univariate analysis, as well as clinically relevant covariates, were entered into the multivariate Cox regression model using the Forward Stepwise method, and adjusted hazard ratios (HRs) and corresponding 95% confidence intervals (CIs) were reported. Statistical significance was established at *p* < 0.05 (two-tailed). All the analyses were performed using IBM SPSS Statistics for Windows, Version 26.0 (IBM Corp., Armonk, NY, USA).

### 2.7. Ethical Considerations

The study was conducted according to the guidelines of the Declaration of Helsinki and approved by the Institutional Review Board ethics committee 13018 del Hospital de Especialidades del Centro Medico Nacional de Occidente R-2023-1301-224 (date of approval 6 November 2023).

Given the retrospective observational design and the use of anonymized clinical data from existing records, the ethics committee formally waived the requirement for informed consent. This study was conducted within the institutional framework for biomedical research and was registered in the IMSS Research Project Database before data collection. Confidentiality and data integrity were strictly maintained throughout the study.

## 3. Results

**Figure 1:**
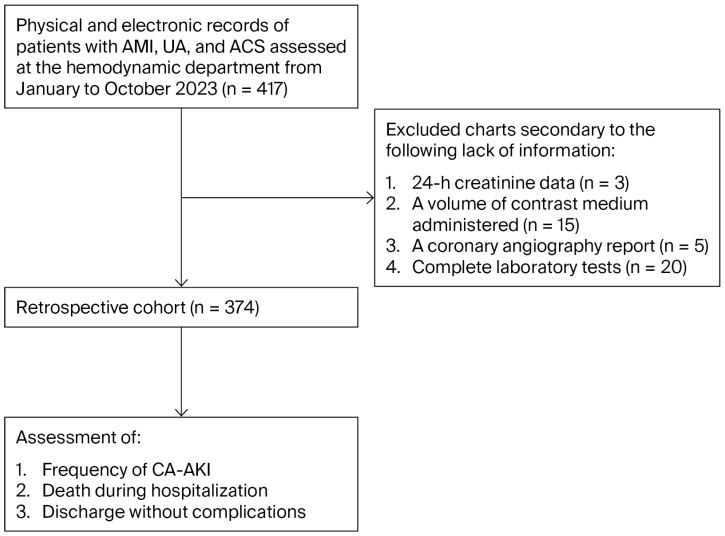
Flow diagram of patient inclusion and selection for the study cohort (January–October 2023). Abbreviations: ACS, acute coronary syndrome; CA-AKI, contrast-associated acute kidney injury.

A total of 417 patients were screened; 43 were excluded due to missing key information—24 h serum creatinine data (n = 3), documented contrast volume (n = 15), a CAG report (n = 5), or complete laboratory test results (n = 20)—resulting in a final analytic cohort of 374 participants with complete follow-up for 30-day all-cause mortality (Figure 1).

The mean age of the cohort was 68.8 ± 11.2 years, and 72.6% were male. As shown in Table 1, the patients who died within 30 days were significantly older than survivors (69.8 ± 9.7 vs. 64.2 ± 11.3 years, *p* = 0.02). The prevalence of traditional cardiovascular risk factors—including hypertension (68.3%), diabetes mellitus (53.8%), and obesity (20.2%)—was high across the cohort, with no significant differences between groups. Notably, delirium during hospitalization was more frequent among non-survivors (9.1% vs. 1.8%, *p* = 0.005), suggesting an association between acute neurological dysfunction and adverse outcomes. Laboratory parameters revealed that the patients who died had higher baseline and post-procedural leukocyte counts, serum glucose, and urea levels, alongside lower eGFR values, all consistent with systemic inflammatory and renal stress states. Importantly, CA-AKI occurred in 17.4% of the patients. These findings support the study’s main hypothesis that CA-AKI, cardiogenic shock, SH, and delirium were independently associated with increased 30-day mortality after CAG.

Table 2 summarizes the univariate analysis of the demographic, clinical, and biochemical variables associated with 30-day mortality. Among the entire cohort, age >60 years was significantly associated with higher mortality (RR, 1.51; 95% CI, 0.64–3.71; *p* = 0.37). Although male sex was predominant (72.6%), it was not significantly associated with death (*p* = 0.27), but the patients who developed CA-AKI had a 2.81-fold increased risk of 30-day mortality (HR, 2.81; 95% CI, 1.48–5.33; *p* = 0.002). SH before the procedure was also associated with increased mortality risk (preprocedural HR, 2.88; 95% CI, 1.54–5.38; *p* < 0.001), and delirium before the procedure was associated with increased mortality risk (preprocedural HR, 7.20; 95% CI, 2.40–20.92; *p* < 0.001). By contrast, variables such as diabetes, hypertension, obesity, and procedural characteristics (contrast volume and PCI site) were not statistically significantly associated with mortality. Post-procedural renal parameters, including urea and eGFR < 50 mL/min/1.73 m^2^, trended toward worse outcomes but were not statistically significant. These findings identify CA-AKI, HS, and delirium as key predictors of short-term mortality following CAG, reinforcing the cardiorenal and inflammatory interplay underlying poor outcomes in this patient population.

**Figure 2:**
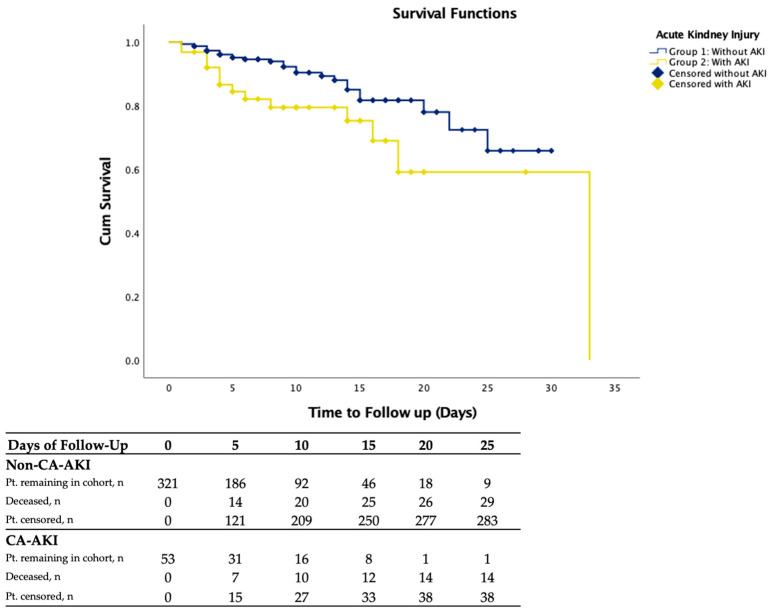
Kaplan–Meier survival curves showing 30-day cumulative mortality in patients with acute coronary syndrome (ACS) undergoing coronary angiography, stratified by presence of contrast-associated acute kidney injury (CA-AKI). Kaplan–Meier survival analysis demonstrating cumulative probability of survival over 30 days following coronary angiography (CAG) in patients with and without contrast-associated acute kidney injury (CA-AKI). The incidence of mortality was significantly higher in the CA-AKI group compared with those without renal injury (log-rank test, *p* < 0.001). CA-AKI was defined as an increase in serum creatinine ≥0.5 mg/dL or ≥25% from baseline within 72 h after contrast exposure, according to the KDIGO 2012 criteria [6]. The shaded areas represent 95% confidence intervals.

Figure 2 illustrates the Kaplan–Meier survival curves for 30-day all-cause mortality, stratified by the presence of CA-AKI. The patients who developed CA-AKI exhibited a markedly lower survival probability compared with those without renal injury (log-rank test, *p* < 0.001). The cumulative 30-day survival rate was 85.1% in the CA-AKI group versus 94.7% in patients without CA-AKI, and in univariate Cox regression, CA-AKI was associated with a 2.32-fold increase in mortality risk (HR, 2.32; 95% CI, 1.23–4.34; *p* = 0.05).

## 4. Discussion

In this retrospective cohort of 374 adults with ACS undergoing CAG, CA-AKI occurred in 17.4% of the patients, and 30-day all-cause mortality was 11.7%. Mortality was substantially higher among the patients who developed CA-AKI (36.4%). In multivariable Cox models, CA-AKI (HR = 2.32; 95% CI: 1.23–4.34), delirium (HR = 3.52; 95% CI: 1.12–11.08), cardiogenic shock (HR = 3.63; 95% CI: 1.76–7.47) and SH (HR = 2.83; 95% CI: 1.42–5.65) were independent predictors of early mortality. These findings support a multisystem vulnerability phenotype in which renal injury, neurocognitive dysfunction, and metabolic stress jointly identify patients at high short-term risk after CAG.

CA-AKI is a common complication following CAG, but its precise definition remains controversial. Some authors have evaluated different diagnostic definitions, noting that it is a frequent complication regardless of the diagnostic criteria used [15,16], and some authors have described the prognosis of patients undergoing CAG who develop AKI during the procedure. Sielski J et al., in a retrospective study, observed that non-radial (femoral) access increased the risk of contrast-induced nephropathy CIN (OR = 2.06 [1.37–3.08]; *p* < 0.001), and the risk-of-death patients with ACS following CAG showed a history of stroke (OR 4.9; 95% CI: 1.58–15.51) and CIN (OR 5.54; 95% CI: 2.49–12.79) [15]. However, that interesting study did not include other relevant factors such as acute metabolic derangement (hyperglycemia) and acute brain dysfunction (delirium). These two factors have been observed to be predictors of bad outcomes in these patients. Lui et al. in a retrospective study described the prevalence of CA-AKI as being 11.2 to 13.0% (adjusted odds ratio [aOR] = 1.38; 95% CI: 1.13–1.68; *p* value < 0.01, *p* for trend < 0.01); 178 patients (13.7%) with AKI died during a standardized follow-up of one year [18]. Other authors have described preoperative conditions as risk factors for CA-AKI before CAG, such as heart failure (HF) and low left ventricular ejection fraction (LVEF < 40%) (OR = 0.852; *p* = 0.031) that were observed by Xu T et al. in a retrospective study [19]. Lun Z et al., in a retrospective cohort study of patients undergoing CAG, used the KDIGO definition for CA-AKI, observing a prevalence of early AKI (32.7%) and late AKI (17.7%). In the mortality analysis using Cox regression, they observed that early CA-AKI (HR = 1.33; 95% CI: 1.02–1.74; *p* = 0.038) was significantly associated with mortality, while late CA-AKI (HR = 0.92; 95% CI: 0.65–1.31; *p* = 0.633) was not [20]. In the Cox regression mortality analysis, early CA-AKI was identified as an independent predictor of long-term mortality (adjusted HR = 1.8; 95% CI: 1.1–2.8; *p* = 0.015), and cardiogenic shock was also identified as a significant predictor of early CA-AKI (aOR = 2.3; 95% CI: 1.1–4.9; *p* = 0.03) [21].

In our center, preprocedural SH was a risk factor for mortality (HR = 2.88; 95% CI: 1.54–5.38; *p* < 0.001). SH or a hyperglycemic state in non-diabetic individuals may be associated with ACS; this hyperglycemia (blood glucose > 180 mg/dL, measured at any time) results from a series of hormonal alterations: (a) increased levels of counter-regulatory hormones to insulin (glucagon, cortisol, catecholamines, and growth hormone); (b) a systemic inflammatory response; and (c) activation of endothelial NADPH oxidase-2 (NOX2) in response to high glucose, which alters the balance between Raf/MAPK-dependent vasoconstriction and PI3K/Akt-dependent vasodilation in favor of constriction. These changes are responsible for the increases in gluconeogenesis, hepatic glycogenolysis, and peripheral insulin resistance that characterize carbohydrate metabolism during stress [22]. Shan Y, in a cross-sectional study in patients undergoing CAG patients, associated the stress hyperglycemia index (SHI) with CA-AKI, observing in the Poisson regression analysis a statistically significant correlation between the lowest and highest levels of fasting SHI and a higher incidence of CA-AKI [(SHI < 0.7 vs. 0.7 ≤ SHI < 0.9), β = 1.828, 95% CI: 1.34–2.48, *p* < 0.001]; [(SHI ≥ 1.3 vs. 0.7 ≤ SHI < 0.9), β = 2.896, 95% CI: 2.08–4.01, *p* < 0.001] [23]. Li Y et al. observed that dysglycemia is closely related to the occurrence of CA-AKI [24]. Other authors have observed in logistic regression analysis that SH was an independent risk factor for coronary artery disease (OR = 4.047; 95% CI: 2.137–7.663; *p* < 0.001) [25]. Zhao Y et al. observed that higher SHI levels were independently associated with an increased risk of CA-AKI (OR = 2.36; 95% CI: 1.56–3.57; *p* < 0.0001), and a J-shaped relationship was observed, with a sharp increase in the risk of CA-AKI when IRS exceeded 1.06. IRS was also a predictor of higher 30-day mortality (*p* < 0.0001) [26]. Zhang Y et al. observed in a retrospective study that patients with multivessel coronary artery disease who presented with a high HRS had a higher risk of cardiovascular mortality after adjustment for confounding factors (high HRS vs. medium HRS: aOR = 1.809; 95% CI: 1.160–2.822; *p* = 0.009) at 5-year follow-up [27]. Armillotta M et al., in a prospective multicenter study, observed a relationship between the presence of type 4 acute myocardial infarction and stress hyperglycemia during percutaneous coronary intervention in diabetic and non-diabetic patients with non-ST-segment elevation acute myocardial infarction. In our center, stress hyperglycemia is a factor associated with mortality that should be further investigated as a trigger for other complications. In our case, it can be associated with acute kidney injury and delirium [28].

Liu S et al. observed in a retrospective cohort study during a control CAG an association between SH and progression of non-target coronary lesions in the patients who underwent PCI with stent placement (OR = 2.12; 95% CI: 1.30–3.44; *p* = 0.003). They observed that SH is a solid factor of progression of non-target coronary lesions in non-diabetic (OR = 3.76, *p* = 0.007) compared with diabetic patients (OR = 1.69; *p* = 0.083) [29]. This prognostic value was confirmed in continuous-variable analyses (HR = 1.59; 95% CI: 1.27–2.00; OR = 3.50; 95% CI: 1.47–8.32 per unit increment) [30].

Our study offers a distinct perioperative approach, integrating hemodynamic and inflammatory variables that are seldom addressed in prior Mexican research. A novel and relevant finding in our cohort was the presence of delirium, which occurred in 9.3% of non-survivors. Multivariate analysis identifies preprocedural delirium as a risk factor for mortality (RR = 7.20; 95% CI: 2.40–20.92; *p* < 0.001). Delirium has emerged as a marker of systemic stress, sympathetic activation, and inflammation, and is increasingly associated with adverse renal and cardiac outcomes [31]. This observation highlights the need for neurocognitive vigilance in perioperative anesthetic management of cardiac patients. Jäckel M et al. described in a retrospective study the incidence of delirium as 10% in patients with acute myocardial infarction who underwent CAG in the intensive care unit. The presence of delirium was associated with increased length of stay in the intensive care unit and hemodynamic instability [32]. The incidence and risk factors for delirium in people with AMI after primary PCI have not been determined. Hayakawa H et al. observed delirium in 27.7% during hospitalization in a retrospective study of patients undergoing PCI. These patients had longer hospital stays, but no differences were observed in in-hospital mortality [33]. Park DY et al. observed a 2.6% prevalence of delirium in a retrospective study of patients undergoing PCI. The patients who developed delirium were older and had more comorbidities, experienced higher in-hospital mortality (aOR = 1.27; *p* = 0.002), and were more likely to be discharged to a facility other than their home (aOR = 3.17; *p* < 0.001). Delirium was also associated with a higher likelihood of intracranial hemorrhage (aOR = 2.49; *p* < 0.001), gastrointestinal bleeding (aOR 1.25; *p* = 0.030), need for blood transfusion (aOR = 1.52; *p* < 0.001), acute kidney injury (aOR = 1.62; *p* < 0.001), and falls in the hospital (aOR = 1.97; *p* < 0.001) [34]. Tan JF et al. observed in a retrospective study that the one-year mortality rate was higher among elderly patients with acute AMI and delirium (17.1% vs. 8.45%, *p* = 0.027); delirium also independently prolonged the stay in the coronary intensive care unit, and the total length of hospital stay [35].

Wang WH, the prevalence of CA-AKI was 16.4% and there was a higher in-hospital mortality rate (15% vs 2.5%, *p* < 0.001). In the Cox regression analysis, the factors associated with mortality were Killip stage 3–4 (HR 2.61; 95% CI 1.27–5.36) and AKI stage 2–3 (HR 3.58; 95% CI 1.97–6.49) [36]. In our center, we observed a similar prevalence of CA-AKI (17.4%). In the Cox regression analysis, the variables associated with mortality were age (HR 1.04; 95% CI 1.00–1.07), CA-AKI (HR 2.81; 95% CI 1.48–5.33), stress hyperglycemia ≥180 mg/dL (HR 2.88; 95% CI 1.42–5.65), and delirium (HR 7.20; 95% CI 2.40–20.92). These observations suggest that mortality in these patients may be related to an inflammatory process secondary to ischemia/reperfusion, which is probably induced by dysregulation of the central autonomic network and activation of the hypothalamic–pituitary–adrenal axis which is currently underdiagnosed.

Limitations of the study: Retrospective cohort studies present limitations inherent to the design, as they are observational studies whose main source of information is medical records; therefore, there is a risk of error and/or bias in the information obtained related to exposure, and the potentially missing relevant information; one example of relevant information that was missing in this study the findings of left ventricular fraction ejection that is an important risk factor related with the outcomes. Other missing variables that were not assessed in our study included, e.g., Killip class and troponin level. Another limitation was that this information is derived from a single center, limiting its external validity (generalizability), and therefore, is applicable to settings with similar characteristics to our center.

This study has several strengths, including other risk factors with limited assessed by other studies such as hyperglycemia and delirium that were included in the same model with CA-AKI in a 30-day follow-up, identifying their relevance as predictors of mortality in a multivariable survival analysis.

Further prospective multicenter studies in Mexico and Latin America are warranted to confirm these associations and test preventive strategies tailored to regional healthcare resources. Future investigations should incorporate emerging biomarkers such as neutrophil gelatinase-associated lipocalin (NGAL) [37], kidney injury molecule-1 (KIM-1) [38], and cystatin C [39], which allow earlier detection of renal stress and may refine risk stratification. Evaluating the impact of anesthesiologist-led hemodynamic management on the incidence of CA-AKI represents a promising direction for applied perioperative research.

In summary, age, CA-AKI, SH ≥180 mg/dL, and delirium were independent predictors of 30-day mortality in ACS patients undergoing CAG. These findings reinforce the need for multidisciplinary peri-procedural strategies that prioritize renal protection, metabolic surveillance, and neurocognitive vigilance in this high-risk population.

## 5. Conclusions

In this retrospective cohort of adults with acute coronary syndrome undergoing CAG, CA-AKI occurred in 17.4% of the patients and was independently associated with higher 30-day all-cause mortality. In addition, SH ≥ 180 mg/dL and delirium were strong independent predictors of early mortality, supporting the concept that short-term risk after coronary angiography in ACS reflects a multisystem vulnerability phenotype rather than an isolated renal complication.

These findings support a risk-stratified peri-procedural approach that prioritizes (1) renal protection and structured post-contrast creatinine surveillance, (2) early identification and management of clinically significant hyperglycemia, and (3) proactive delirium detection and prevention strategies. Prospective multicenter studies in Mexico and Latin America are warranted to confirm these associations and to test pragmatic care bundles targeting renal, metabolic, and neurocognitive complications to reduce early mortality after coronary angiography in ACS.

## Figures and Tables

**Table 1:** Comparison of characteristics between patients who died vs. survived after coronary angiography.

Variable	Total (n = 374)	Death (n = 44)	Survival (n = 330)	*p*
Sociodemographic and anthropometric data				
Age, years	68.82 ± 11.22	69.84 ± 9.74	64.17 ± 11.25	0.02
Sex: Male, n (%)	270 (72.6)	34 (79.1)	236 (71.7)	0.31
IMC, kg/m^2^	27.36 ± 5.57	27.58 ± 4.82	27.34 ± 4.54	0.75
Habits				
Tobacco use, n (%)	231 (62.1)	28 (65.1)	203 (61.7)	0.74
Alcohol consumption, n (%)	170 (45.7)	16 (36.4)	155 (47.1)	0.18
Drug use, n (%)	22 (5.9)	0	22 (6.7)	0.08
Comorbidities				
Systemic arterial hypertension, n (%)	256 (68.3)	34 (77.3)	222 (67.3)	0.18
Type 2 diabetes mellitus, n (%)	200 (53.8)	25 (58.1)	175 (53.2)	0.63
Obesity, n (%)	75 (20.2)	7 (16.3)	68 (20.7)	0.50
Previous events of heart attack, n (%)	119 (32)	18 (40.9)	102 (30.9)	0.18
Dyslipidemia, n (%)	86 (22.8)	6 (13.6)	79 (23.9)	0.13
Chronic kidney disease, n (%)	37 (9.9)	5 (11.6)	32 (9.7)	0.69
Delirium, n (%)	10 (2.7)	4 (9.1)	6 (1.8)	0.005
Chronic obstructive pulmonary disease, n (%)	10 (2.7)	2 (4.7)	8 (2.4)	0.39
Asthma, n (%)	5 (1.3)	0	5 (1.5)	0.42
Admission diagnosis				
STEMI, n (%)	191 (51.1)	26 (59.1)	165 (50)	0.26
NSTEMI, n (%)	52 (14)	8 (18.2)	43 (13.9)	0.45
Unstable angina, n (%)	52 (14)	1 (2.3)	51 (15.5)	0.02
Other diagnoses *, n (%)	36 (9.7)	6 (13.6)	35 (10.6)	0.55
Angiographic characteristics				
Contrast medium volume, mg iodo/mL	170.74 ± 83.65	171.98 ± 94.62	170.58 ± 82.23	0.92
Percutaneous coronary intervention, n (%)	292 (78.1)	30 (68.2)	262 (79.4)	0.09
Site of PCI, n (%)				
Left anterior descending artery, n (%)	125 (34.4)	12 (27.3)	113 (34.2)	0.36
Right coronary artery, n (%)	93 (24.9)	12 (27.3)	81 (24.5)	0.69
Circumflex artery, n (%)	57 (15.20)	8 (18.2)	49 (14.8)	0.56
Triple-vessel disease, n (%)	69 (18.4)	8 (18.2)	61 (18.5)	0.96
Without coronary occlusion	30 (8)	4 (9.1)	26 (7.9)	0.77
Total occlusion, %	76.54 ± 27.76	73.72 ± 30.55	76.91 ± 27.4	0.48
Duration of hospital stay, hours	203.10 ± 147.58	188.65 ± 149.50	204.98 ± 147.42	0.50
CA-AKI, n (%)	65 (17.4)	16 (36.4)	49 (14.8)	<0.001
Laboratory pre-coronary angiography				
Hemoglobin, g/dL	14.11 ± 2.32	14.56 ± 2.72	14.05 ± 2.27	0.17
Anemia (Hb < 9 g/dL)	4 (1.2)	0	4 (1.2)	1.00
Leukocytes/μL	10.78 ± 4.43	13.47 ± 6.10	10.42 ± 4.03	0.003
Leukocytosis (>11,000 cells/μL)	156 (41.7)	25 (56.8)	131 (39.7)	0.03
Platelets/μL	246.40 ± 84.14	239.72 ± 82.03	247.37 ± 84.49	0.58
Thrombocytopenia (<100,000 platelets/μL)	4 (1.2)	1 (0.3)	3 (0.9)	0.40
Creatinine, mg/dL	1.27 ± 1.46	1.46 ± 0.76	1.24 ± 1.52	0.37
Creatinine >1.5 mg/dL	61 (16.3)	16 (36.4)	45 (13.6)	0.001
Glucose, mg/dL	154.71 ± 84.43	188.09 ± 101.29	150.35 ± 81.14	0.02
Glucose (>180 mg/dL)	89 (23.8)	20 (45.5)	69 (20.9)	0.001
BUN, mg/dL	44.69 ± 28.84	56.21 ± 27.34	43.32 ± 28.77	0.006
BUN (>40 mg/dL)	163 (43.6)	30 (68.2)	133 (40.3)	<0.001
eGFR, mL/min/1.73 m^2^	90.65 ± 14.25	82.42 ± 12.05	91.70 ± 14.18	<0.001
eGFR (<50 mL/min/1.73 m^2^)	6 (1.6)	0	6 (1.8)	1.0

Continuous variables are expressed as mean ± standard deviation (SD) and categorical variables as number (percentage). Statistical comparisons were performed using Student’s t test or the Mann–Whitney U test for continuous variables and the chi-square or Fisher’s exact test for categorical variables, as appropriate. *p* < 0.05 was considered statistically significant. Abbreviations: BMI, body mass index; BUN, blood urea nitrogen; eGFR, estimated glomerular filtration rate; PCI, percutaneous coronary intervention; LVEF, left ventricular ejection fraction; CA-AKI, contrast-associated acute kidney injury; ACS, acute coronary syndrome. * Other diagnoses: Minoca syndrome and Wellens syndrome.

**Table 2:** Univariate analysis of clinical, biochemical, and procedural factors associated with 30-day mortality in patients with acute coronary syndrome (ACS) undergoing coronary angiography.

	Intro Method			Stepwise Method		
Variable	HR	95% CI	*p*	HR	95% CI	*p*
Age ≥60 years	1.51	0.64–3.71	0.37	-	-	-
Age (years)	1.03	1.00–1.07	0.04	1.04	1.00–1.07	0.03
Male, n (%)	1.62	0.72–3.63	0.27	-	-	-
Alcohol consumption, n (%)	0.73	0.35–1.39	0.31	-	-	-
Previous heart attacks, n (%)	1.49	0.80–2.7	0.20	-	-	-
Delirium, n (%)	5.76	1.93–17.21	0.002	7.20	2.40–20.92	<0.001
Unstable angina, n (%)	0.14	0.01–1.10	0.06	-	-	-
Complications						
CA-AKI, n (%)	2.02	1.10–4.40	0.002	2.81	1.48–5.33	0.002
Preoperatory laboratory						
Hyperglycemia (≥180 mg/dL)	2.58	1.31–5.09	0.006	2.88	1.54–5.38	<0.001
Leukocytosis (≥11,000 cells/μL)	0.64	0.33–1.23	0.18	-	-	-
Thrombocytopenia	2.64	0.33–21.13	0.36	-	-	-
eGFR, mL/min/1.73 m^2^ (baseline)	0.97	0.96–0.99	0.04	-	-	-

Qualitative variables are expressed as frequency (%). Hazard ratios (HRs) and 95% confidence intervals (CIs) were calculated using univariate analysis. Continuous variables were dichotomized according to clinically relevant cutoffs (age > 60 years, glucose > 180 mg/dL, leukocytes > 11,000 cells/μL, eGFR < 50 mL/min/1.73 m^2^). *p* < 0.05 was considered statistically significant. Abbreviations: CA-AKI, contrast-associated acute kidney injury; eGFR, estimated glomerular filtration rate; NSTEMI, non-ST-segment elevation myocardial infarction; STEMI, ST-segment elevation myocardial infarction.

## Data Availability

The data presented in this study are available from the corresponding author upon reasonable request. Due to institutional and ethical restrictions related to patient privacy, the datasets are not publicly available; de-identified data may be shared upon approval by the institutional review board and in accordance with IMSS data governance policies.
